# Fit for LIFE: the development and optimization of an intervention delivered through prison gymnasia to support incarcerated men in making positive lifestyle changes

**DOI:** 10.1186/s12889-022-13004-3

**Published:** 2022-04-18

**Authors:** Alice MacLean, Matthew Maycock, Kate Hunt, Craig Mailer, Keith Mason, Cindy M. Gray

**Affiliations:** 1 Institute of Social Marketing and Health, University of Stirling, Stirling, UK; 2 School of Education and Social Work, University of Dundee, Dundee, UK; 3 Institute of Social Marketing and Health, University of Stirling and Institute for Health and Wellbeing, University of Glasgow, Glasgow, UK; 4 HMP Perth, Perth, UK; 5 HMP Kilmarnock, Kilmarnock, UK; 6 Institute of Health and Wellbeing, School of Social and Political Sciences, University of Glasgow, Glasgow, UK

**Keywords:** Prisoner health, Health behaviour change, Prison, diet, Healthy eating, Physical activity, Weight, Sedentary behaviour, Wellbeing

## Abstract

**Background:**

Despite prison settings presenting opportunities for healthy eating and regular exercise, many incarcerated men supplement prison food with unhealthy snacks and drinks, and are less likely to achieve recommended physical activity guidelines than non-incarcerated men. This paper describes the co-development with prison staff of a healthy lifestyle intervention for delivery to incarcerated men, and feasibility testing of its delivery through prison physical education departments.

**Methods:**

The starting point for intervention development was Football Fans in Training (FFIT), an evidence-based intervention successful in engaging men and supporting them to lose weight, make positive lifestyle changes and maintain these long term. We iteratively tested and adapted FFIT for delivery in prison gym facilities through a four Phase pilot and optimisation study. Methods used to evaluate each phase included: observations of session deliveries; semi-structured interviews with participants; and a focus group/semi-structured interviews with prison Physical Education Instructors (PEIs) who delivered the programme. Data were analysed thematically using the Framework approach. Findings from each phase informed development of the optimised programme.

**Results:**

We iteratively co-developed a healthy lifestyle intervention (known as Fit for LIFE) tailored to the needs of incarcerated men and prison operational constraints. Fit for LIFE comprises elements specifically designed to address common barriers to a healthy lifestyle within prison, including: discussion of healthiest available food choices; trying out different physical activity options in the prison gym; and strategies (such as in-cell workouts) for dealing with prolonged time in cells at evenings/weekends. Weight loss was not always the most valued outcome. Instead, participants cited a wide range of behavioural, physical and mental health improvements as important to them, and were more motivated if they could focus on identifying and achieving personally relevant objectives.

**Conclusions:**

Fit for LIFE is a 10-week, group-based healthy lifestyle programme tailored for delivery to incarcerated men in prison gymnasia. Weekly 90-min sessions include informative and interactive ‘classroom’ activities followed by a practical physical activity training session, often with group activities. Fit for LIFE aims to help incarcerated men to: increase physical activity; reduce sedentary time; eat more healthily; and start and maintain using prison gym facilities with confidence.

**Supplementary Information:**

The online version contains supplementary material available at 10.1186/s12889-022-13004-3.

## Background

People who are incarcerated often have poorer physical and mental health than the general population [1–3]. Prison settings have the potential to support improvements in the health and wellbeing of these marginalised groups, in part through promotion of healthier lifestyles [4]. Prison meals are provided in line with current dietary recommendations [5] and for many prisoners represent an improvement on their diet before entering prison [6]. However, a recent Scottish Prison Service (SPS) survey found only 61% were content with the menu choice [7], with most prisoners supplementing prison meals with less healthy food and snacks (e.g., sugar-sweetened drinks, biscuits, chocolate, crisps) from the weekly ‘canteen’ (prison shop) delivery 1.

In addition, despite having opportunities to undertake physical activity outdoors in the exercise yard and prison gymnasium, people who are incarcerated are less likely to achieve recommended physical activity guidelines than those not in prison [8]. Almost half (48%) of SPS prisoner survey respondents reported not using the gym at all, and only 43% reported achieving at least 30 minutes of moderate exercise (e.g., brisk walking) ≥5 times a week [7]. Qualitative research reports how extended time in cells leads to people who are incarcerated becoming increasingly sedentary and unfit, and contributes to them becoming “emotionally unstable, psychologically disturbed and socially dislocated” [3 , p.123].

Football Fans in Training (FFIT) is a community-based intervention that has proved to be successful in helping men who are overweight and at high risk of ill-health to lose weight, improve their diet and physical activity, and maintain these changes long term. FFIT uses the appeal of the football club to attract men aged 35–65 years with BMI ≥ 28 kg/m^2^ to attend 12 weekly sessions delivered by club community coaches at club home stadia [9]. FFIT is delivered by community coaching staff at professional football clubs to groups of up to 30 men, and was specifically designed to work with, rather than against, prevailing conceptions of masculinity, whilst also taking account of best evidence in weight loss and behaviour change [9]. The programme is ‘gender-sensitised’ in relation to context (the traditionally male environment of football clubs and men-only groups), content (information on the science of weight loss presented simply – ‘science but not rocket science’) and style of delivery (participative, peer-supported learning and positive male ‘banter’ to facilitate discussion of sensitive subjects). Each weekly session combines a ‘classroom component’ providing advice on healthy eating and/or use of behaviour change techniques (e.g., self-monitoring, goal setting) with coach-led practical physical activity training. Participants also follow an incremental pedometer-based walking programme to try to incorporate physical activity into their daily lives [9].

A randomised controlled trial conducted in 2011/12 demonstrated FFIT’s effectiveness, cost-effectiveness and success in engaging men from all socioeconomic groups [10]. At 12-month follow-up, the mean between-group weight loss difference was 4.94 kg (95% CI 3.95-5.94, p<.0001) in favour of the intervention group [11]. Significant benefits were also seen for secondary outcomes, including improvements in self-reported physical activity and diet (intake of fatty and sugary foods and fruit and vegetables), as well as positive changes in self-reported psychological health (self-esteem, positive and negative affect and physical health-related quality of life). At 3.5 year follow-up, intervention group men weighed 2.9kg less than at baseline (~1/3 maintained weight loss ≥5%) and had sustained improvements in physical activity, diet and many psychological outcomes [12].

In 2012, the FFIT research team at the University of Glasgow was approached by a Physical Education Instructor (PEI) from one of the prisons (co-author CM) asking if FFIT could be adapted for delivery to male prisoners within the prison gym setting. This paper therefore describes the process of working in partnership with two Scottish prisons to iteratively build on the FFIT model to co-develop a healthy lifestyle group-based intervention for incarcerated men (known as Fit for LIFE) and assess the feasibility of its delivery by PEIs through prison physical education departments.

## Methods

We conducted a pilot and optimisation study over four phases in which we iteratively tested and adapted the FFIT programme for delivery in prison gymnasia. In the two prisons which participated in the study, available facilities within the gymnasia included a weights/cardiovascular area, a sports hall, outdoor pitches and changing areas. As part of the study, we also assessed the feasibility of collecting data on a range of clinical (including weight, body mass index, waist circumference, blood pressure, self-reported medication use), behavioural (including self-reported physical activity, diet, smoking and sleep) and psychological (including self-esteem, motivation for exercise, self-efficacy for behavioural change, aggression and wellbeing) outcomes and obtaining fasting blood and urine samples. We do not report these data here.

**Fig. 1:**
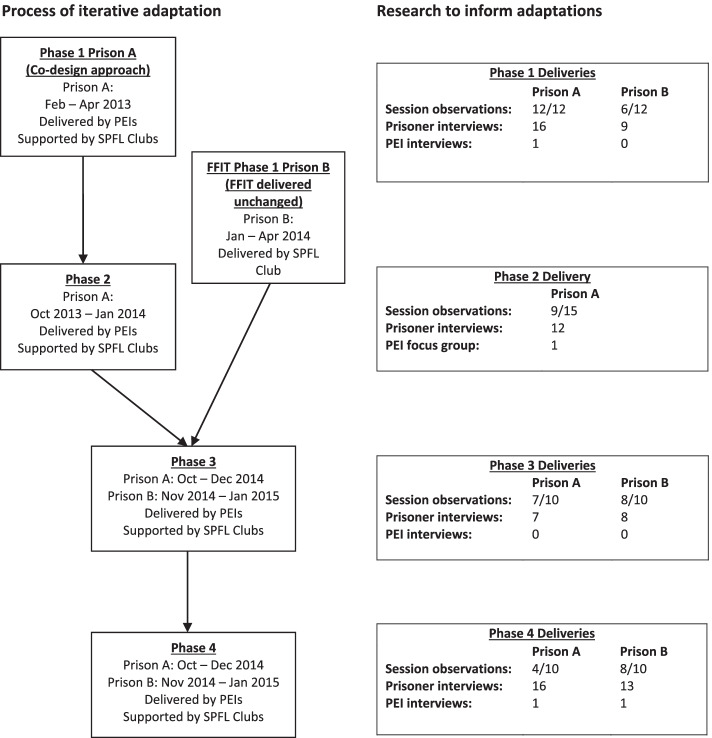
Schematic overview of the development of the Fit for LIFE programme

An overview of the process of developing the Fit for LIFE intervention is provided in Fig. 1; it shows each of the four phases of intervention testing and adaptation alongside details of the research conducted to inform intervention development. Phase 1 involved testing two different models of tailoring FFIT for delivery in the prison setting. We took a co-design approach to adapt FFIT for delivery by PEIs. At Prison A, this involved consulting with the PEIs, prison governor and head of offender outcomes initially, and then more intensively with the PEIs at several meetings prior to the delivery of Phase 1. Subsequently at both prisons, PEIs were asked to provide feedback during and at the end of programme delivery at each phase. In addition, after considering participant engagement (through session observation data) and feedback (collected in one-to-one post-programme interviews) at the end of each phase, the research team specifically consulted PEIs about the proposed changes for the next phase.

Prior to the delivery of Phase 1, we identified the target group as incarcerated men (18+ years) with BMI > 27kg/m^2^ who were not using the prison gymnasium. It was agreed that for Phase 1 minimal changes would be made to the key components of the 12-week FFIT programme [9]. We worked with the PEIs to make initial minor adaptations that took account of the constraints of the prison context and made the materials (PEI delivery manual and participant notes) more applicable to the new target population. For example, we took particular care to make the participant notes accessible for men with low literacy [13] and removed or changed content which could not be usefully applied within the prison context (e.g. the suggestion of taking the dog for an extra walk as a way of increasing physical activity). As was the case in the FFIT programme, participants were given pedometers and encouraged to record step-counts daily while following a plan to increase steps incrementally as a way of incorporating physical activity into their daily lives and developing their self-monitoring skills. PEIs received a half-day training in the programme delivery protocol from the research team and a health expert who co-developed the FFIT training for football club coaches. The Phase 1 programme was delivered in Prison A between February and April 2013 with two local Scottish Professional Football League (SPFL) coaches each supporting one session. PEIs recruited participants to the programme, mainly by approaching individuals directly in the prison residential halls (to facilitate engagement with those not using the prison gymnasium) and word of mouth.

After we had started working with Prison A, we learned that another establishment (Prison B) had secured NHS funding and independently approached their local SPFL club to lead a delivery of FFIT in Prison B. This provided an opportunity to compare a ‘naturally-occurring translation’ of FFIT into the prison setting with the programme we were co-designing with PEIs at Prison A. This Phase 1 programme in Prison B was delivered over 12 weeks between January and April 2014 by two coaches from the local SPFL club supported at each session by PEIs. To recruit participants to the programme, PEIs approached men on the prison halls, particularly those not using the prison gym facilities, and also displayed posters in communal areas. Prisoners trained in health improvement were also involved in this programme, specifically in encouraging men to sign up and then to attend each week.

Process evaluation data were collected during and after programme delivery, and used to inform iterative programme redevelopment and further deliveries in Phases 2-4 until the findings suggested the programme format and content had been optimised. As Fig. 1 shows, the methods used to evaluate each phase included: observations of programme sessions; semi-structured interviews with participants (both those who completed and those who were identified by PEIs as not completing the programme); and a focus group/semi-structured interviews with PEIs delivering the programme.

### Observations of programme sessions

The observations involved audio-recording and detailed notetaking of each programme session with written consent from the PEIs and all participants. All members of the research team were involved in the observational data collection, although no more than two were present at any one session, and most sessions were observed by only one researcher. In Phase 1 at Prison A, observations were shared between CMG, KH and AMacL. Phase 1 sessions at Prison B were observed by MM, CMG, KH and AMacL. MM conducted observations for all other phases, with AMacL, KH and CMG attending at least one session. The researcher(s) took written notes during the sessions and/or dictated further reflections into a digital recorder immediately afterwards. The digital reflections were transcribed and fieldnotes written up electronically with a focus on: the extent to which PEIs adhered to the delivery protocol; group-based factors (e.g., group dynamics, participants’ levels of engagement with ‘classroom’ and physical activity content each week); and identification of examples of particularly good practice and/or problems/issues (see also [14]).

### Participant interviews

Semi-structured one-to-one participant interviews were also conducted to explore views on the acceptability of the programme and its component activities, what worked well or not so well, and any changes that should be made. The interviews were conducted immediately after the end of the programme deliveries. Interviews were chosen rather than focus groups for participants as this better suited the logistical constraints of the prison environment. It also ensured that individual prisoners were given the opportunity to voice their opinions without more vocal group members dominating or group discussions going off topic. During Phase 1 at Prison A, a convenience sampling approach was taken whereby participant interviews were conducted by KH, CMG and AMacL with as many participants as were available and willing to be interviewed (including those who had not completed the programme) at times when the researchers visited the prison to collect process evaluation data. Interviews took place in the gym and small meeting rooms in the residential halls, with prison staff nearby but not able to overhear what participants were saying. During Phase 1 at Prison B and subsequent phases in both prisons, MM conducted participant interviews in the same way. Participant interviews were audio-recorded with interviewees’ written consent and transcribed verbatim.

### PEI interviews and focus group

PEI interviews (Phase 1 and 4) and one focus group (Phase 2) were conducted to explore their views on what worked well or not well, and to gather suggestions for changes that should be made to subsequent iterations of the programme. A similar convenience sampling approach as that used with participants was adopted. Interviews were conducted in the PEI office space within the gymnasia. A focus group was conducted after Phase 2 delivery at Prison A with the PEIs who had delivered the programme and were available on the day that CMG and KH were visiting the prison for data collection. The focus group took place in a prison meeting room. The PEI interviews and focus group were audio-recorded with written consent and transcribed verbatim. Informal discussions with PEIs at both prisons (rather than formal data collection) were used at the end of Phase 3 to finalise the changes for the Phase 4 deliveries.

Rather than aiming for data saturation, we were able to triangulate the data from session observations, participant interviews, and the PEI interviews and focus group to ensure consideration of reflections from a variety of viewpoints and compare the different perspectives in order to refine the programme at each phase.

### Data analysis

Transcripts were checked for accuracy against original recordings and anonymised by removing identifiable information. All were analysed thematically using the Framework approach [15] with NVivo 10 software assisting data organisation, coding and retrieval. MM and AMacL conducted a first stage of transcript familiarisation and analysis independently. To ensure analytical rigour and consistency, consultation then took place with CMG and KH who had reviewed a sub-sample of the transcripts. Based on these discussions, and informed by our main research questions, a coding frame was devised which captured: acceptability of/satisfaction with the programme content; participants’ motivations to sign-up; reasons for leaving/continuing; issues specific to the prison-context; reflections on programme components (positive/negative comments, helpful/unhelpful elements); suggestions for changes/additions; and group dynamics and relationships. The coding frame was applied to the remaining transcripts by MM. This approach also allowed for identification and systematic exploration of emergent and unanticipated themes.

Extracts from the interviews, focus group and session observations are presented below and labelled to indicate participant or PEI ID (e.g., Participant 14, PEI 1), whether participants had completed the programme or not (completer/non-completer), phase in which the programme was being delivered (Phase 1, Phase 2, Phase 3, Phase 4) and prison (Prison A, Prison B). The data extracts presented have been selected because they best illustrate the points being made and represent the views expressed. All extracts have been changed from local dialect to standard English.

## Results

### Phase 1 deliveries

The 12-week Phase 1 programme at Prison A was completed by 13 (68%) of the 19 men who enrolled. Details of how many participants were present at the first and final session of each phase at both prisons are provided in the supplementary material (Appendix 1, Table A1).

Participant interviews suggested that most enjoyed the sessions. When asked if there was anything he did not like about the programme, one said:

No, there was nothing that I didn’t like about it. I liked it every week. I only missed one week and that’s ‘cause got a tooth pulled and they told me not to do anything strenuous in case the wound opens. So, I only missed one session like but nah, I loved it.

**Participant 9 (completer), Phase 1, Prison A**

Another said that he was motivated to attend most weeks by “just the involvement, just to see everybody and see how they’re doing and things like that, learning new things as well about how to live” (**Participant 11 (completer), Phase 1, Prison A**).

Many spoke about making positive changes to the health behaviours targeted by FFIT (diet and physical activity), but a few also spoke of changing other health behaviours. For example, some described how the programme had encouraged them to cut down or stop smoking^2^:

Through the programme... I’ve been trying to stop smoking for ages … I stopped smoking. And, my health is getting better, you know, my blood pressure’s went down

**Participant 5 (completer), Phase 1, Prison A**

PEIs reported that a broad range of outcomes were important to participants, including eating and/or sleeping better, improved self-esteem and wellbeing and improved physical fitness (e.g., strength, flexibility, endurance). The PEIs themselves particularly valued the fact that the programme had succeeded in attracting men who were not previously using the prison gym facilities and had encouraged them to start attending. However, one suggested the programme’s focus on weight loss was off-putting for some participants:

Although they made gains in lots of other areas, the fact of we sort of put up here [emphasised] weight-loss, what it was all about, and at week sort of 6, 7 [when participants were re-weighed], some of them didn’t really feel they’d made a lot of gains there, and lost a bit of momentum [after that]

**PEI 1, Phase 1, Prison A**

This concern was also highlighted in the Phase 1 Prison A programme observation field notes:

Quite a lot of [the participants] hadn’t lost that much weight, but one thing that has been emphasised throughout the programme is the fact that you should take out of the programme what you want, and that might not necessarily be weight loss. […] a couple of the guys perhaps aren’t losing as much weight as they might have hoped to and, perhaps, are finding this a little dispiriting.

**Week 6 observation fieldnotes, Phase 1, Prison A**

The PEIs suggested that more opportunities for one-to-one discussion would help participants identify personally relevant objectives (e.g., in relation to physical activity, diet or weight loss) and support needs. They also reported that participants were talking about the programme and what they were learning from it with their families during visiting times, and this was seen as a valuable outcome for the men. This view was echoed by participants:

It was an insight for my girlfriend too, I’ve been explaining to her as it went along and I need to get some of the paperwork [participant notes] photocopied to send out to her, explain to her about like the portion size and the sugars and salts and things in foods, know what I mean.

**Yeah, so you’ve been talking with her about that when she’s come into visit you?**

Yeah. She was pretty shocked at it [some of the information on portion size, etc.] as well, know what I mean?

**Participant 11 (completer), Phase 1, Prison A**

During the Phase 1 programme at Prison A, a number of challenges specific to the prison context were evident. One of these was that many participants were eager to spend the majority of each session doing physical activity rather than on the classroom component. For example:

…the first three weeks, or something, we were doing plenty like, we got to go and like basically do plenty... fitness stuff, you know? We were able to use the gym, you know, and all that caper, and then we just sort of started getting like […] you were talking about how to lose weight, and then twenty minutes of actually losing weight, do you know what I’m on about? […] If there was more activity in it, I thought it would’ve been a bit better, you know. Like obviously more... maybe an hour and twenty minutes doing activities, and then the last forty-five minutes, thirty minutes you know talking about losing weight, or what we’d learnt […] But, there was none of that…

**Participant 16 (non-completer), Phase 1, Prison A**

The PEIs also commented on the need to make the classroom component shorter to keep participants engaged:

We can get better at delivering the [classroom component]. The reason being they’ve not got an attention span that [they] can sit for forty minutes, and if they can get a hold of something that’s nothing at all to do with the course [programme], like a wee bugbear […] they can take you away on a tangent […] So we’ve got to get quicker at delivering [the classroom component] if we could get it down to a half hour window of opportunity, get the main points over…

**PEI 1, Phase 1, Prison A**

Participants’ complaints about prison meals were another context-specific challenge that arose. Observation field notes captured the ways in which PEIs initially dealt with participants’ claims that it is difficult to eat a healthy diet in prison:

[The PEIs] tried to keep bringing it back [from participants complaining about prison food], for the men to see the bigger picture and not always to be thinking about [food] in relation to the prison. So, if [participants] would say things like “yeah, but there’s no, you don’t get a veg option in the prison”, [the PEIs] would be saying “well, for one, I think you do always have a veg option, but for another, you know, try and think about this as wider, you’re not going be in here forever. We want you to think about this for your lives outside of the prison. We want you to be thinking about feeding this back to your family and to your children”. So that was interesting, [the PEIs] were trying not to get bogged down in […] complaining too much about prison food…

**Week 2 observation fieldnotes, Phase 1, Prison A**

Some participants explained how dissatisfaction with prison meals led to them buying additional (generally unhealthy) food from the prison canteen list:

Even the PEIs know that the food [in prison] isn’t that great, you know? But you just, you need to work around it, make do with what you’ve got, and all that, but it’s easier said than done, ‘cause if you’re going to lose weight you can’t eat the jail food […] And, if you don’t eat the jail food you’ve got to buy stuff off the canteen, smoked sausage, pastas... and that’s all that’s there. The stuff that you can cook, Pot Noodle, and all that, it’s full of fat […] preserved crap, you know.

**Participant 14 (non-completer), Phase 1, Prison A**

Another context-specific challenge to maintaining a healthy lifestyle which emerged during the participant interviews was having to spend extended periods locked up in their cells at weekends. Many blamed lockups for contributing to their unhealthy snacking:

...it’s boring [in your cell]. When you’re not working or doing like a course or anything, you, all you’ve got is your own mind, you know what I mean? And it’s quickly, you’re bored quickly [...] Once your door shuts, you’ve got a telly, radio or smoking and eating. [Plus] you’ve got a box full of stuff from the canteen.

**Participant 7 (completer), Phase 1, Prison A**

Along with buying unhealthy foods and snacks from the canteen, it became apparent that prior to taking part in the programme, participants were also purchasing large amounts of sugar-sweetened beverages. However, this was a habit that some said they found relatively easy to change:

I was bad for fizzy juice, I was drinking a lot of fizzy juice, like probably ten litres from like a Friday to the other Wednesday [...] about two litres a day [...] Well, I’ve stopped that altogether. [...]

**What are you drinking instead then?**

Just diluting [juice]. Sugar free. [...] And coffees [...] cut right back on that as well.

**And did you take a lot of sugar [in the coffee]?**

Just taking one sugar, [before I took] two, or three.

**Participant 4 (completer), Phase 1, Prison A**

The Phase 1 findings also revealed how a wide range of circumstances led some participants to be absent from sessions or drop out altogether. These included unexpected transfers to other prisons; early release; work, health and legal appointments; illness, injury and other life events. Interviews with non-completers revealed how life stressors (e.g., concerns about release or family issues on the outside) could impact negatively on their motivation to continue with the programme:

I had problems outside with my daughter […] And, I just – my heart just went out of it [the programme] basically. […] I liked [the programme]. It was just with things going on, and that. I like – if I’m going to do something I like to be fully committed to do it.

**Participant 13 (non-completer), Phase 1, Prison A**

Another non-completer revealed that he dropped out of the programme because he did not get on with other participants:

I'd like to do it again when it came up, eh? It's just the boys that was on it, they... it's like I said, remember the other boy […] The older guy. Remember when the first day we did it, he fell over when we were...? […] Laughing at him and everything as well and I just didn’t think it was right, eh? […] If you couldn’t do something, they would laugh at you and that, and you could see them blatantly laughing at you. But... I couldn’t really say... go and say anything in front of everybody to them and that because it would just end up in fighting in here. […] I thought either come out o’ the course or say something to them and perhaps end up fighting, and so I thought “Right, I'll just come out of the course and maybe get a chance to do it again in the future.”

**Participant 7 (non-completer), Phase 1, Prison A**

At Prison B, the Phase 1 delivery of FFIT led by local SPFL football club coaches was completed by 10 (66%) of the 15 men who enrolled. Observations showed that the SPFL football coaches, who had no prior experience of working with men in prison, initially found the group interactions very challenging. In an attempt to deal with these challenges, they deviated from the FFIT delivery protocol and spent most of each session delivering group physical activity, including outdoor football games, and typically condensed ‘classroom’ activities into a few minutes at the end of the sessions. As a result, many of the core components of the FFIT programme were not delivered; and although participants were largely positive about the programme (“I don't actually think there was anything I would change because I loved it” **Participant 4 (completer), Phase 1, Prison B**), some felt the ‘banter’ between the men and coaches sometimes became negative or out of control:

Maybe just some of the carry-on was a wee bit maybe a bit much. They [other participants] could have took it a bit more seriously.

**Participant 11 (completer), Phase 1, Prison B**

One participant suggested greater PEI involvement might ensure the programme’s sustainability:

I thought they [PEIs] would then participate in the kind of Fat Club [colloquial name for the FFIT programme amongst participants in Prison B] then run it on and carry it on. Not just watch, then when it’s finished, that’s it finished. Until somebody else comes in and ‘let’s do another course’. Where they could have watched it, took a bit from it, and maybe started another course themselves.

**Participant 2 (completer), Phase 1, Prison B**

### Phase 2 programme

The Phase 1 findings at Prison A informed the development of the Phase 2 programme which was delivered only at Prison A. Table 1 summarises the main adaptations made for the Phase 2 delivery. Changes to the programme *format* included: extending its length from 12 to 15 weeks to reduce the length of the classroom component to approximately 30-35 minutes in each session and to add a ‘graduation’ ceremony to celebrate men’s participation and achievements at the end of the course. The opportunity to take participants aside during the physical activity sessions for one-to-one or small group objective-setting/goal review discussions was added to better support them to achieve their individual behaviour change goals and to discuss ways of overcoming any difficulties they were facing in making changes.

**Table 1:** Summary of the main changes for the Phase 2 programme

Change Made	Rationale
*Programme format*	
Programme extended from 12 to 15 weeks	To promote participant engagement by reducing time in classroom activities in each session and include graduation ceremony
One-to-one/small group discussion activities added during physical activity sessions	To support participants to achieve their goals, and help those requiring additional support
*Programme content*	
*Reduction of emphasis on weight loss through:*	
Individual discussion of personal objectives	To promote motivation throughout the programme by ensuring participants have objective(s) they value and can achieve
Introduction of beep, strength and stretch tests in Weeks 1, 8 and 14	To provide concrete evidence of improvement in physical activity and fitness levels
Inclusion of monitoring of progress in relation to wellbeing and sleep	To encourage participants to recognise and value these outcomes
*Increased emphasis on sedentary behaviour*	
Measurement and feedback on sedentary behaviour via activPAL^TM^ at start and end of programme	To illustrate how much time participants spend sitting (and when), and to provide evidence of any changes made
Focus on breaking up sedentary behaviour at weekend (e.g., inclusion of ‘in-cell workouts’)	To help participants identify ways of being active while locked in cells at weekends
*Physical activity*	
Inclusion of physical activity of participants’ choice	To promote a sense of ownership of the programme around a valued positive behaviour among participants
*Diet*	
Focus on avoiding unhealthy eating at weekend (e.g., ‘weekend coping strategies’)	To help participants identify how to reduce unhealthy snacks while locked up at the weekend
Specific discussion of how to make healthier choices from prison menus and canteen lists	To help participants identify ways of improving their diet within the constraints of prison meal provision
Physical representation of sugary drinks	To promote participant engagement in reducing sugary drinks intake
*Addition of smoking cessation*	
Discussion of quitting smoking	To provide participants who want to reduce or stop smoking with an opportunity to be supported in doing so
*Social support*	
More inclusion of team physical activity team activities, and fun competition promoted (including the superstars programme)	To promote a sense of belonging among participants and enhance engagement and social support
Inclusion of a former participant or passman at each session	To provide peer support
Inclusion of visitor notes	To encourage men to talk about their participation in the programme and information about leading a healthier lifestyle at family visits
*Promoting sustained changes*	
Introduction of relapse prevention strategies (if-then plans)	To provide (and help participants practice) a technique for avoiding setbacks
Reflections on the benefits of change	To promote a sense of wellbeing
Discussion of sustainability of change according to men’s sentences (e.g., long term, imminent release, release within 12 months)	To encourage personally-relevant discussion of how to maintain changes made

Adaptations were also made to the programme *content*. Reducing the emphasis on weight loss was achieved by including a focus on improving physical activity and diet in the individual or small-group discussions to agree personally relevant objectives. Beep, strength and stretch tests were added in weeks 1, 8 and 14 to provide participants with concrete evidence of their improvement in physical activity and fitness levels, an outcome which the Phase 1 programme had suggested many particularly valued. To help men recognise their progress each week, simple emoticons (happy and sad faces) were added to their personal weekly progress record together with a ‘How am I feeling?’ text box inviting them to reflect on changes in their wellbeing.

A number of activities were introduced to help participants address the challenges posed by long periods locked up in their cells at weekends. First, they were given the opportunity to wear a device to monitor their sedentary behaviour (activPAL^TM^) at the start and end of the programme to provide them with objective feedback on how much time they spent sitting (and when), as well as evidence of any changes they had made while taking part in the programme. Second, ‘in-cell workouts’ (exercises participants could do in their cells) aimed to help them identify ways of breaking up sedentary time and alleviating boredom while locked up. Third, a discussion of ‘weekend coping strategies’ was included to help participants identify ways of reducing their consumption of unhealthy snacks when locked in their cells for longer periods.

A specific focus on how to make healthier choices from prison menus and canteen lists was added to help participants identify ways of improving their diet within the perceived constraints of prison food provision. Physical representation of the amount of sugar in soft drinks aimed to encourage participants to reduce their intake of sugar-sweetened beverages. Discussion of smoking cessation was also included to provide those wanting to reduce or stop smoking with an opportunity to be supported in doing so.

A number of changes were made to enhance social support. A former participant or gym passman^3^ was invited to attend each session to provide peer support during the programme. Participants were also offered a copy of the programme notes to give to their visitors if they wished to discuss the healthy lifestyle changes they were making during family or other visits. To further enhance social support and engagement, additional team activities and fun physical activity competitions were included, and participants were invited to suggest the types of physical activities they particularly enjoyed during these group-based sessions.

Finally, given evidence that setbacks (e.g., over-eating/being sedentary at weekends, life stressors, etc.) were a major problem throughout the programme, a number of activities were introduced to enhance behaviour change maintenance once the programme had finished. These included: relapse prevention strategies (such as ‘if-then plans’ [16] in Week 13) to provide participants with techniques for avoiding setbacks; reflection on the benefits to them of the changes they had made; and specific discussion of how to sustain change in relation to their prison sentences (e.g., long-term, imminent release, release within 12 months).

The Phase 2 programme was delivered at Prison A from October 2013 to January 2014 and 16 participants were enrolled. Attendance varied across the 15 weeks and fell towards the end: only 5 (31%) participants attended Week 14. However, when interviewed, participants were generally positive about the programme and described multiple benefits from participating, including improved eating habits (fewer sweets/biscuits, more fruit), weight loss, feeling fitter and feeling motivated to attend gym sessions:

I was overweight, and I didn’t get a lot of enthusiasm about going to the gym [before doing the programme], and I needed to get a kick up the arse to be, just to get myself back. For me, it’s got me back to doing gym again. It’s got me off my arse and back into the frame of mind of being in the gym and doing a full workout in the gym.

**Participant 3 (completer), Phase 2, Prison A**

The visitor notes were enthusiastically received; some men said the programme became a positive focus for family visits:

I passed it [visitor notes] on to my family as well, know, through it, know? Well, I told the wife and that, know what I mean, that I was on it […] and it gave us something to talk about at visits […] telling her what we’d done in the class and that.

**Participant 9 (non-completer), Phase 2, Prison A**

A common complaint was that there was still too much classroom content (for example, most interviewees thought the smoking cessation discussion, which was added for the Phase 2 delivery, was unnecessary/irrelevant). Some felt that a 15-week programme was too long and that some sessions dragged:

“The little talks at the start were okay […] The football and everything was fine. That was fine. There was a few weeks it was stagnant. It drifted away in the middle.” **Participant 6 (completer), Phase 2, Prison A**

Observation fieldnotes highlighted some additional issues with the Phase 2 programme. Some participants complained about skin irritation from wearing the activPAL^TM^ activity monitor (which was affixed to the thigh for seven days), and others disbelieved the feedback on sedentary behaviour it provided. A Phase 1 participant, who had been invited to attend all Phase 2 sessions to provide social support to the participants, was a useful role model initially. However, he became more disruptive as the programme progressed and needed reminding that he was there to support new participants, not simply to take part in the programme himself. Finally, participants did not want to give up time doing physical activity for one-to-one or small group discussions, and as a result these did not happen.

### Phase 3 programme

Findings from the Phase 2 programme at Prison A and the Phase 1 programme at Prison B were used to develop the Phase 3 programme which was delivered at both institutions. Table 2 summarises the main changes made for Phase 3, many of which aimed to simplify and reduce the programme content. First, the overall length of the programme was shortened from 15 to 10 core weeks to promote engagement. Second, information which was not directly relevant to participants’ current lives (e.g., role of alcohol in weight gain) or not considered to be a key activity (e.g., smoking cessation) was removed to encourage participants to engage with the key messages they could act on directly. Third, as provision of activPAL^TM^ feedback on sedentary behaviour had proved problematic, this element was removed.

**Table 2:** Summary of the main changes for the Phase 3 programme

Change Made	Rationale
*Programme format*	
Programme reduced from 15 to 10 core weeks (plus individual enrolment and end of programme measurement sessions, and graduation)	To promote engagement throughout the programme, particularly towards the end
One-to-one and small group work became less formalised	Participants did not want to take part in structured ‘classroom’ activities during physical activity session
*Programme content*	
Reduced by removing information not directly relevant to participants at that time (e.g., role of alcohol in weight gain)	To keep classroom sessions shorter despite reduced length of programme, and to encourage participants to engage with key messages they could act on directly
activPAL^TM^ feedback removed	Provision of activPAL^TM^ feedback was problematic and would not be sustainable for post-research roll-out and scale up
Smoking cessation removed	Most Phase 2 participants said that stopping smoking was not their focus
*Physical activity*	
Discussion of gym sessions and classes available introduced in Week 2	To encourage participants to try out and build the habit of attending the gym while they were on the programme
*Social support*	
Former participant, passmen or health champion involvement recommended, with clarification of their role in supporting delivery rather than simply attending and taking part in the programme	To provide peer support during and between sessions, and motivate participants to attend sessions
*Maintenance of change*	
Setbacks introduced earlier (Week 4) and then revisited throughout the programme	Setbacks are a major problem throughout the programme, therefore introducing them earlier and more frequently, will support participants to avoid or overcome them

In addition, to encourage participants to build the habit of using the gym facilities during and after the programme, a discussion of the gym sessions and classes that were available outside programme sessions was included early in the programme (in Week 2). Clarity was also provided about the role of any former participant or passman attending the programme, specifically that they were there to support delivery rather than simply take part in sessions, and more flexibility recommended (e.g., their attendance was not required at all sessions).

At Prison A, the Phase 3 programme took place from October to December 2014 and 16 men enrolled. Each session was attended by over half of participants, with 9 (56%) attending Week 10. At Prison B, the programme was delivered from November 2014 to January 2015; however, detailed weekly attendance was not available due to logistical challenges. Observations at both institutions suggested good engagement from participants and that the shortened programme length was appropriate. Flexible peer support, particularly involving passmen to assist in delivering some components, worked well.

At Prison B, the local football club did not attend any sessions, despite being invited. To fill this gap and retain the function of having support from external agencies to bring novelty and added value, the PEIs invited coaches from other local sports organisations to some sessions. At Prison A, an external tennis coach delivered one session. Participants at both institutions were positive about this change of focus away from football, as not all the participants liked football.

Sedentary behaviour emerged as a continued problem at both institutions, particularly at weekends:

[Taking part in the programme] has changed, aye it’s changed a lot, man. Don’t get me wrong, as I said, I still have wee blowouts now and again, like…[…] Like ‘cause see on a Saturday and a Sunday you’re dubbed up [locked in your cell] at like six o’clock. And then on a Saturday, I need to stay up ‘til six o’clock in the morning, so that’s twelve hours I’m sitting in my cell, just so I can watch a UFC [Ultimate Fighting Championship] that finishes at six in the morning.

**Participant 5 (completer), Phase 3, Prison B**

Nevertheless, despite the challenges (e.g., during weekend lockups), participants still felt that sedentary behaviour was something they could change. One participant described how attending the programme had made him realise that, for him, boredom and sitting too much had become a vicious circle:

I think because I wasn’t going to bed ‘til one o’clock in the morning and then you know, you were tired during the day, you couldn’t be bothered walking round and round anyway. You were quite comfortable sitting, which was pretty poor, and you just… Mm hmm. Just laziness. But it’s what, you know, a lot of people struggle with these things. Uh huh. But I think particularly in prison it is quite difficult.

**Participant 1 (completer), Phase 3, Prison A**

Another participant referred to his pedometer as having a positive impact on his physical activity at weekends:

With the stepometer [sic], trying to better what you’ve done. Weekends forced you to, ‘cause they’d be lazy days for me. Especially a Sunday, I’ve only averaged maybe two or three thousand [steps]. But I’ve got off my arse and maybe got ten thousand or whatever.

**Participant 4 (completer), Phase 3, Prison A**

As in Phases 1 and 2, levels of attendance continued to be impacted by factors beyond participants’ control, as Participant 6 at Prison A explained:

I was on remand for [nearly a year]. So, it was a year of my life not knowing what was going to happen and then your class [Fit for LIFE] started and I was in the middle of my case. That’s why I wasn’t there some weeks […] I had QCs, I had psychiatrists, I had everybody coming up and it was always on your day [when the programme was delivered]. It’s not as if I make the appointments […] it was just the way it was happening. And like the [other participant], he was in the same hall as me and he’s saying, “Are you going?” And I’m like, “No, I’ve got this this week, I’ve got that this week.” It was 4 weeks in a row that I didn’t make it […] And then I got sentenced and then I started coming again.

**Participant 6 (completer), Phase 3, Prison A**

### Phase 4 programme

Minor refinements for the Phase 4 programme were informed by the Phase 3 findings at both prisons. As shown in Table 3, even more emphasis was placed on reducing sedentary behaviour throughout the programme, and local sports coaches were invited to support some sessions instead of football club coaches. This broadening of focus to include other sports reflected the fact that, in addition to problems in obtaining commitment from local football clubs, the PEIs felt it would be beneficial, for both the programme (given some participants’ preferences for other sports) and the prison gym in general, to build links with other local sports organisations.

**Table 3:** Summary of the main changes for the Phase 4 programme

Change Made	Rationale
*Programme content*	
Increased emphasis on reducing sedentary behaviour, particularly in Weeks 4 and 7	This is a behaviour that participants continue to identify as problematic and amenable to change
*Physical activity*	
Inviting local sports coaches to attend sessions instead of FFIT football club coaches	Not all participants were interested in football, and engagement from local professional football clubs was often difficult to secure. The PEIs also felt it would be beneficial both to extend links to other local sports organisations

The Phase 4 programme took place at Prison A from September to December 2015, where 18 men enrolled, and from November 2015 to January 2016 at Prison B. At Prison A, at least half of participants attended each session and 9/18 (50%) attended Week 10. Once again, weekly attendance data were not available at Prison B. At both institutions, unexpected transfers to other institutions, early release, work and other commitments (e.g., health/legal appointments), illness, injury and other life events, including those occurring to family members on the outside, continued to lead to absences and dropouts. However, informal PEI feedback highlighted that levels of attendance and engagement with Fit for LIFE were comparatively higher than previous programmes they had delivered.

In general, the Phase 4 programme was positively received, despite some negative comments (particularly from non-completers) about the classroom component and about the physical activity sessions being too intense in early weeks, or not structured enough:

The theory was good but they’ve kind of overdone it every week [...] The only thing I would really...that would’ve kept me going, like I say, would be more [physical] activity, more structured [physical] activity.

**Participant 13 (non-completer), Phase 4, Prison A**

Participants reported improvements in diet, physical activity (including increased use of prison gym facilities) and weight. In addition, the novelty of undertaking a group-based programme in the gym, and the resultant positive dynamics both within the group and between participants and the PEIs, were valued by some men:

The guys that were on it and the guys that were doing it. Uh huh. It was a brilliant atmosphere. There was no hassle, no fighting or that, no carry-on. It was just – came in, got a good laugh.

**Participant 13 (completer), Phase 4, Prison B**

The Phase 4 findings suggested that optimisation of the programme had been achieved. An outline of this final version of the Fit for LIFE programme is provided in Table 4. A summary of the contents of each session is provided in the supplementary material (Appendix 2, Table A2).

**Table 4:** The optimised Fit for LIFE programme

**What is Fit for LIFE?** A 10-week, structured, manualised group-based health behaviour change programme tailored for delivery to incarcerated men. Weekly, 90-minute sessions are delivered by PEIs in prison gym facilities and include informative and interactive ‘classroom’ activities followed by a practical physical activity training session, often with group-based activities. Although no formal post-programme support is provided, it is emphasised during delivery that PEIs will be available to provide participants with ongoing support and advice about their physical activity, diet and weight, as long as they remain in the establishment. **What are the aims of Fit for LIFE?** To help participants to: increase physical activity; reduce sedentary time; eat more healthily; and use prison gym facilities on a regular basis, during the programme and after it finishes. **What does the Fit for LIFE dietary component entail?** An emphasis on the importance of eating a healthy balanced diet, both to attain and maintain a healthy weight, and to help participants feel better by having more energy. Participants are encouraged to identify sustainable changes they can make and set detailed, achievable goals which will enable them to reduce: consumption of foods high in fat and sugar; portion sizes; and sugar in tea/coffee, and increase consumption of fruit, vegetables and wholemeal bread. **What does the Fit for LIFE physical activity component entail?** The importance of increasing physical activity and reducing sedentary behaviour is emphasised. Participants are shown how to gradually incorporate more physical activity into their daily routine through an incremental pedometer-based walking programme. Weekly group sessions include a physical activity component delivered using prison gym facilities. Participants are shown how to build up their activity levels gradually and get to experience a range of different physical activities in the gym so they can find an activity that they enjoy and can continue post-programme. **What makes Fit for LIFE suitable for delivery within the prison context?** The dietary and physical activity components of the programme comprise elements specifically designed to address common barriers to a healthy lifestyle within the prison context. These include: discussion of healthiest choices on prison menu/canteen lists; strategies for increased time in cells at evenings/weekends (e.g., how to sit/lie less and eat more healthily); and demonstration of in-cell workout options.	

## Discussion

This paper reports the co-development of the Fit for LIFE programme that aims to help incarcerated men increase physical activity, reduce sedentary time, eat more healthily, and increase their use of prison gym facilities. Developed in close partnership with prison staff and with input from participants in Fit for LIFE sessions, the programme content and format were carefully adapted for the setting and target population over four iterations. The co-development process ensured that the delivery of the final 10-week programme was feasible within the operational constraints of two very different high security prisons (one state, one private) and acceptable to prison staff and the men taking part in Fit for LIFE.

Although the highly successful FFIT programme [9, 11, 12] was the starting point for Fit for LIFE, substantial modifications over multiple iterations were required before achieving an optimised programme for delivery to men in high security prisons^4^. The length of the programme and the time spent within sessions on ‘classroom activities’ was reduced as much as possible while still ensuring that the essential programme content could be delivered. This was done to facilitate participant attendance levels, which were affected by other appointments and commitments, and to recognise participants’ shorter attention spans and desire to spend as much time as possible doing physical activity.

Instead of focussing on weight loss as the main intervention outcome, participants were supported to set individual healthy lifestyle objectives which reflected their own circumstances. There was increased focus on ways to break up sedentary behaviour, particularly when locked in cells for extended periods at weekends, and increased emphasis on setbacks and relapse prevention to reflect the chaotic nature of participants’ lives and the competing demands on their attention. The optimised programme was less focussed on football to reflect the fact that not all participants were football fans and to encourage the development of links between prisons and other community sports organisations. Several elements were added to facilitate peer support, and to encourage participants to talk about the programme and elicit support from their families during visits. For example, a former Fit for LIFE participant was invited to take part in the programme to provide support and encouragement; and participants were offered an extra copy of programme materials to pass on to family or other visitors to encourage discussion about the lifestyle changes they were trying to make. Lastly, a graduation ceremony was added to the final week of the programme to celebrate and acknowledge participants’ achievements.

The ‘active ingredients’ of FFIT that were essential to retain and adapt to the prison context included key behaviour change techniques such as goal setting, self-monitoring and problem solving. Additionally, as the interactive nature of FFIT is essential to its success, it was important to ensure that Fit for LIFE participants felt part of a group of men who were like them and that they were given opportunities to bond as a group through taking part in physical activity together. Evidence on the development and evaluation of health and social interventions for use in prisons is sparse. However, there are some parallels between Fit for LIFE and other programmes designed for use with incarcerated men. One study assessing the impact of an exercise intervention on incarcerated men’s mental health outcomes reported wide-ranging benefits for participants, including improved routine and socialisation (through having a reason to leave their cell), and improved self-esteem, mood and self-reported general health [17]. Our qualitative research yielded similar positive results, with Fit for LIFE participants and the prison staff delivering it reporting various ways in which participation in the programme had contributed to positive health behaviour changes and improvements in participants’ mood and motivation levels.

A review of parenting programmes for male young offenders reported that interventions were generally conducted in groups, were information-based and designed to encourage interaction and discussion between participants with low literacy levels, and were viewed positively by participants and prison staff [18]. However, the authors noted that often the information provided lacked relevance for participants, and that more tailoring of intervention components was needed to take account of and address the challenges posed to participants both in prison and on their release. This tailoring of the intervention to the constraints of the prison context is something which was a particular focus during the development of Fit for LIFE.

Finally, Buston [19] describes the development and implementation of a parenting intervention for young incarcerated fathers and suggests that one of the reasons for its success in recruiting, retaining and engaging participants was because they had many things in common, which created a sense of shared identity and facilitated interactions among the men. Despite these high levels of engagement, this study also reported that events beyond participants’ control, such as court appearances, health appointments and unexpected transfers or release, had a negative impact on retention. Similarly, our study found that the unpredictable and highly changeable nature of participants’ lives meant it was challenging to maintain high levels of attendance and engagement.

Following the optimisation of Fit for LIFE, a sustainable model for future delivery and monitoring and evaluation was developed in partnership with SPS staff. A two-day Train-the-Trainer package was designed to facilitate rollout of Fit for LIFE across the SPS, which began in 2017. Currently, programme deliveries are continuing across all prisons in Scotland. Fit for LIFE has also been delivered to young offenders and incarcerated women. For example, staff facilitating a pilot delivery of Fit For LIFE in a women-only prison were largely positive about the programme^5^. Finally, the PEIs involved in the co-design process (CN and KM) were commended for their roles in developing and implementing the Fit for LIFE programme in the 2017-18 Butler Trust Awards, which recognise and celebrate outstanding work and best practice across UK prisons, probation and youth justice.

### Strengths and limitations

The co-design approach was essential to the success of this model of programme development. Indeed, it was one of the PEIs (CM) who first took the initiative to approach the FFIT research team to ask whether the programme could be adapted for delivery within a prison setting. A crucial aspect of the co-design was orientation at the early stages of programme development to the challenges and constraints specific to the prison environment gained through consultation with the PEIs and senior management (e.g., prison governor, head of offender outcomes). Being given the opportunity to develop and pilot programme iterations in two very different prison settings is a further strength of the study, as is the fact that the work undertaken builds on and utilises the skills and experience of PEIs in working with people in custody.

This study also has some limitations. Although we highlighted the need for private and quiet spaces to conduct participant interviews, this was not always possible to arrange. Some interviews had to be conducted within the PEI office in the prison gym, which resulted in frequent interruptions and may have had an impact on the conversations that took place in those interviews. The recording quality also suffered due to background noise from the adjacent gym hall. The sample of participants who took part in interviews was self-selected; however, considerable efforts were made to interview as many as possible, including those who had dropped out of the programme. It could be argued that participants may have been reluctant to voice negative opinions to the researchers conducting the interviews, given they knew the researchers had also developed the programme. However, the frequency of researcher visits to the programme helped to create a rapport with participants, and this enabled participants to offer positive and negative feedback both during the session observations and in post-programme interviews. Furthermore, the collection of data from three sources facilitated the triangulation of perspectives, allowing for consideration and comparison of reflections on the programme from a variety of viewpoints.

At both prisons, the PEIs led recruitment, using a mixture of talking to men individually about the programme and posters in communal areas. Once men had committed to attending the programme, the PEIs then invited them to attend the first session of Fit for LIFE (Phase 1) or baseline measurements (all other phases). This approach did not allow us to estimate the numbers of eligible men who were invited to participate, and therefore we are unable to report response rates or compare response rates for each recruitment strategy.

## Conclusions

Fit for LIFE is a structured group-based healthy lifestyle programme for delivery by prison PEIs to incarcerated men in prison gymnasia. Ten weekly 90-minute sessions include informative and interactive ‘classroom’ activities followed by a practical physical activity session using gym facilities. In developing Fit for LIFE using a co-design approach, we have shown that it is possible to develop a structured healthy lifestyle intervention for incarcerated men, although several iterations were required to tailor the programme to their needs and prison operational constraints. We found the Fit for LIFE programme was successful in attracting men who were not previously using prison gym facilities to what is often perceived to be a more positive space within the prisons. This brought the men into regular contact with the PEIs, promoted positive relationships, as well as health behaviour change and increased use of gym facilities, and provided opportunities for them to perform more positive forms of masculinity [20, 21].

## Supplementary Information

**Additional file 1.** Supplementary Materials.

## Data Availability

Due to the sensitive nature of the data collected in this study, they are not suitable for sharing. The Fit for LIFE delivery manual and participant notes are available on request from the corresponding author.

## Abbreviations

BMI: Body mass index
CI: Confidence interval
FFIT: Football fans in training
NHS: National health service
PEI: Physical education instructor
SPFL: Scottish professional football league
SPS: Scottish prison service

## Footnotes

1 Prisoners can purchase food, drinks and other sundry items from the prison canteen list each week.

2 At this time, smoking was still permitted within certain areas of prisons in Scotland and around 70-75% of people in custody were smokers [6]. Scotland’s prisons became smoke free on 30 November 2018.

3 Passmen are prisoners who have additional responsibility in relation to different aspects of the running of the prison. Passmen in the gym help with cleaning and organising the facilities.

4 We also undertook feasibility work with men in an open prison but concluded that it was not feasible to run Fit for LIFE within this context due the complexities caused by men being allowed to spend periods of time at home with their families and attending multiple appointments to prepare them for release. We do not report this work here.

5 The successful pilot did not lead to any further deliveries of Fit for LIFE to women due to wider changes happening across the female prison estate in Scotland.
