# Supplementary material for: Fit for LIFE: the development and optimization of an intervention delivered through prison gymnasia to support incarcerated men in making positive lifestyle changes
Source: BMC Public Health. 2022 Apr 18;22:783. doi: 10.1186/s12889-022-13004-3 (PMC9017016; doi:10.1186/s12889-022-13004-3)
Supplement: Supplementary file 1 — Additional file 1. Supplementary Materials. [file 12889_2022_13004_MOESM1_ESM.docx]

**Supplementary Materials**

**
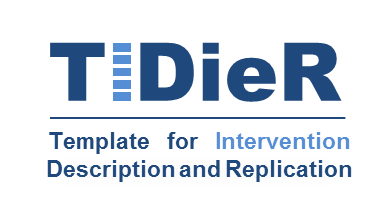
The** **TIDieR (Template for Intervention Description and Replication) Checklist*:**

Information to include when describing an intervention and the location of the information

| **Item no** | **Item** | **Where located **** | |
| --- | --- | --- | --- |
|  |  | Primary paper  (page no) | Other ^†^ (details) |
|  | **BRIEF NAME** |  |  |
| **1.** | Provide the name or a phrase that describes the intervention. | 1_________ | ______________ |
|  | **WHY** |  |  |
| **2.** | Describe any rationale, theory, or goal of the elements essential to the intervention. | 4 & 5______ | _____________ |
|  | **WHAT** |  |  |
| **3.** | Materials: Describe any physical or informational materials used in the intervention, including those provided to participants or used in intervention delivery or in training of intervention providers. Provide information on where the materials can be accessed (e.g., online appendix, URL). | 41________ | Delivery manual and participant notes available on request |
| **4.** | Procedures: Describe each of the procedures, activities, and/or processes used in the intervention, including any enabling or support activities. | 46-47 (Table 6) | _____________ |
|  | **WHO PROVIDED** |  |  |
| **5.** | For each category of intervention provider (e.g., psychologist, nursing assistant), describe their expertise, background and any specific training given. | 6, 8, 26 & 28 | _____________ |
|  | **HOW** |  |  |
| **6.** | Describe the modes of delivery (e.g., face-to-face or by some other mechanism, such as internet or telephone) of the intervention and whether it was provided individually or in a group. | 6 ________ | _____________ |
|  | **WHERE** |  |  |
| **7.** | Describe the type(s) of location(s) where the intervention occurred, including any necessary infrastructure or relevant features. | 6_________ | _____________ |
|  | **WHEN and HOW MUCH** |  |  |
| **8.** | Describe the number of times the intervention was delivered and over what period of time including the number of sessions, their schedule, and their duration, intensity or dose. | 38 (Figure 1) | _____________ |
|  | **TAILORING** |  |  |
| **9.** | If the intervention was planned to be personalised, titrated or adapted, then describe what, why, when, and how. | 7, 39-40 (Table 1) | _____________ |
|  | **MODIFICATIONS** |  |  |
| **10.^ǂ^** | If the intervention was modified during the course of the study, describe the changes (what, why, when, and how). | 12-29, 42-44 (Tables 3-5) | _____________ |
|  | **HOW WELL** |  |  |
| **11.** | Planned: If intervention adherence or fidelity was assessed, describe how and by whom, and if any strategies were used to maintain or improve fidelity, describe them. | 9_________ | _____________ |
| **12.^ǂ^** | Actual: If intervention adherence or fidelity was assessed, describe the extent to which the intervention was delivered as planned. | 12-29_____ | _____________ |

** **Authors** - use N/A if an item is not applicable for the intervention being described. **Reviewers** – use ‘?’ if information about the element is not reported/not sufficiently reported.

† If the information is not provided in the primary paper, give details of where this information is available. This may include locations such as a published protocol or other published papers (provide citation details) or a website (provide the URL).

ǂ If completing the TIDieR checklist for a protocol, these items are not relevant to the protocol and cannot be described until the study is complete.

* We strongly recommend using this checklist in conjunction with the TIDieR guide (see *BMJ* 2014;348:g1687) which contains an explanation and elaboration for each item.

* The focus of TIDieR is on reporting details of the intervention elements (and where relevant, comparison elements) of a study. Other elements and methodological features of studies are covered by other reporting statements and checklists and have not been duplicated as part of the TIDieR checklist. When a **randomised trial** is being reported, the TIDieR checklist should be used in conjunction with the CONSORT statement (see [www.consort-statement.org](http://www.consort-statement.org)) as an extension of **Item 5 of the CONSORT 2010 Statement.** When a **clinical trial** **protocol** is being reported, the TIDieR checklist should be used in conjunction with the SPIRIT statement as an extension of **Item 11 of the SPIRIT 2013 Statement** (see [www.spirit-statement.org](http://www.spirit-statement.org)). For alternate study designs, TIDieR can be used in conjunction with the appropriate checklist for that study design (see [www.equator-network.org](http://www.equator-network.org)).

**Appendix 1**

**Table A1**

**Number of participants present at first and final session of each phase at both prisons.**

|  | **Participants present at first session** | **Participants present at final session** |
| --- | --- | --- |
| **Phase 1** | | |
| Prison A | 19 | 13 |
| Prison B | 15 | 10 |
| **Phase 2** | | |
| Prison A | 16 | 5 |
| Prison B | - | - |
| **Phase 3** | | |
| Prison A | 16 | 9 |
| Prison B | Detailed attendance data not available | |
| **Phase 4** | | |
| Prison A | 18 | 9 |
| Prison B | Detailed attendance data not available | |

**Appendix 2**

**Table A2**

**Content of optimised Fit for LIFE programme**

| *Session* | *Classroom* | *Physical activity* |
| --- | --- | --- |
| Week 1 | Getting started | |
|  | - Introduce men to aim of programme: ‘*how to be more active, sit and lie around less, eat better, feel better and stay that way in the long term’* - Importance of commitment - Importance of self-monitoring | - Introduction to pedometers and how to use them - Baseline step count homework - Beep, strength and stretch tests - Discussion of future activity sessions |
| Week 2 | Getting more active | |
|  | - Personal objectives - Problem with inactivity and sedentary time - Food diary homework | - Pedometer steps - Ways to increase step counts and step count targets - Information about classes/sessions at the gym - Low intensity team activity (e.g., scoring goals, and hitting targets with badminton racquet) |
| Week 3 | **Healthier eating** | |
|  | - Explanation of food groups and eating a healthy diet (fewer fatty/sugary foods, more fruit and vegetables, and where possible, wholewheat bread/pasta and brown rice) - Food diaries and prison menu/canteen compared with healthy eating recommendations - SMART goal setting and action planning introduced | - Step count review - Low intensity team activity (e.g., scoring goals, and hitting targets with badminton racquet) |
| Week 4 | **Weight management and reducing sitting and lying time** | |
|  | - SMART goal review - Health benefits of good weight management - Energy balance - Reducing sitting and lying around - Food label homework | - Demonstration of in-cell workout to try during the week - Aerobic circuit in gym with step count challenge - Step count review |
| Week 5 | **Setbacks and healthier eating** | |
|  | - Stages of change and setbacks - Understanding food labels and choosing healthier foods - Canteen lists and tips for healthy eating - Dealing with setbacks | - Discussion of in-cell workout homework - Aerobic circuit (based on the men’s own suggestions) with some competitive element - Step count review |
| Week 6 | **Halfway down and weekends** | |
|  | - - - - Sitting and lying less at the weekend       - If-then plans       - Importance of social support       - Measurements taken to review progress | - Beep, strength and stretch tests - SMART goal and step count review - Activity of men’s choice |
| Week 7 | **Benefits of physical activity and reducing sedentary time** | |
|  | - Review of progress - Benefits of being more active and less sedentary - Barriers to PA - Inactivity and losing what you’ve gained | - Rate of perceived exertion (RPE) scale - Activity session using men’s suggestions with some element of competition - Step count review |
| Week 8 | **Thinking about eating** | |
|  | - - - - Non-alcoholic drinks and a healthy lifestyle       - Making favourite meals healthier - Damage limitation in prison (menus and evenings/weekends in cell) - SMART goals | - Activity session using men’s suggestions with some element of competition - Step count review |
| Week 9 | **How are we doing and what to look out for** | |
|  | - Reflection on benefits of change - Sustainability of change - Food or other diary homework | - Step count and activity review - Superstars training |
| Week 10 | **Looking back and life after Fit for LIFE** | |
|  | - Feedback of progress - Before and after diaries - On-going SMART goals | - Grand “Superstars” competition |
|  | **Graduation** | |
|  | - A celebration of achievement, possibly including families and other visitors | |
